# Effects of sex hormones on bronchial reactivity during the menstrual cycle

**DOI:** 10.1186/1471-2466-14-108

**Published:** 2014-07-01

**Authors:** Maria Matteis, Francesca Polverino, Giuseppe Spaziano, Fiorentina Roviezzo, Carlo Santoriello, Nikol Sullo, Maria Rosaria Bucci, Francesco Rossi, Mario Polverino, Caroline A Owen, Bruno D’Agostino

**Affiliations:** 1 Department of Experimental Medicine-Section of Pharmacology, 2nd University of Naples, 80136 Naples, Italy; 2 Division of Pulmonary and Critical Care Medicine, Brigham and Women’s Hospital/Harvard Medical School, Boston, MA, USA; 3 Department of Experimental Pharmacology, University Federico II of Naples, Naples, Italy; 4 Cava de’ Tirreni Hospital, Cava de’ Tirreni, Italy; 5 The Lovelace Respiratory Research Institute, Albuquerque, NM, USA

**Keywords:** Perimenstrual asthma, Menstrual cycle, Testosterone, Phosphodiesterases, Cyclic AMP

## Abstract

**Background:**

Many asthmatic women complain of symptom exacerbations in particular periods, i.e. during pregnancy and menstrual cycles (perimenstrual asthma: PMA)". The goal of this study was to study the effect of the luteal and follicular phases of the menstrual cycle on bronchial reactivity (BR) in a group of asthmatic women.

**Methods:**

For this purpose, 36 pre-menopausal women were enrolled and underwent testing for resting pulmonary function, measurement of the diffusing capacity of the lung for carbon monoxide (DLCO), and airway responsiveness to methacholine in the follicular and luteal phases of their menstrual cycles. We also measured plasma hormone levels and levels of cyclic adenosine monophosphate (cAMP; a mediator of bronchial smooth muscle contraction) and testosterone in induced sputum samples.

**Results:**

Our study showed that about 30% of the asthmatic women had decreased PC_20_FEV_1.0_ in the follicular phase of menstrual cycle with a significant correlation between PC_20_FEV_1.0_ and serum testosterone levels. Moreover, marked increases in sputum testosterone levels (mean = 2.6-fold increase) together with significant increases in sputum cAMP concentrations (mean = 3.6-fold increases) were observed during the luteal phase of asthmatic patients, suggesting that testosterone contributes to the pathophysiology of PMA. We excluded the possibility that testosterone directly inhibits phosphodiesterase (PDE) activity as incubating PDE with testosterone in vitro did not reduce PDE catalytic activity.

**Conclusions:**

In conclusion, our data show that PC_20_FEV_1.0_ was decreased in the follicular phase of the menstrual cycle in about 30% of women and was associated with lower cAMP levels in sputum samples, which may contribute to bronchoconstriction. Our results also suggest a link between PMA and testosterone levels. However, whether these findings are of clinical significance in terms of the management of asthma or asthma worsening during the menstrual cycle needs further investigation.

## Background

Asthma is a chronic inflammatory disorder of the airways [1] in which many cells and cellular elements play a role [2,3]. The incidence, severity, and prognosis of asthma can be affected by several factors, including the patient’s age and sex. Epidemiological studies of both incidence and prevalence have reported a male predominance of asthma before puberty and a female predominance after puberty.

Sex differences exist in the risk, incidence, and pathogenesis of various lung diseases in humans [4]. Females typically are more susceptible and/or develop more severe asthma, chronic obstructive pulmonary disease, lung cancer, and other lung conditions. Accumulating epidemiological and experimental data suggest that sex hormones may be important physiological modulators in the lung, and the role of estrogens in asthma has received considerable attention in this regard.

It has been known for a long time that some female asthmatic patients experience an aggravation of asthma symptoms during the premenstrual or menstrual phases of their cycle. In particular, it has been hypothesized that hormonal fluctuations during the menstrual cycle play a significant role in the pathophysiology of asthma, resulting in periodic worsening of disease severity in adult females [5-11]. Terms such as (pre-) menstrual, circamenstrual, or perimenstrual asthma (PMA) have been used to describe this phenomenon. Various approaches have been adopted to investigate the hormonal hypothesis. However, the roles of gender and sex hormones in asthmatic women are complex and not completely understood. A more complete understanding of the activities of hormones in regulating asthma exacerbations could introduce new strategies for symptom management and decrease the disease burden associated with this phenomenon.

Therefore, the goal of this project was to study the effect of the menstrual cycle; specifically, the luteal and follicular phases and plasma sex hormone levels, on bronchial reactivity (BR) in a group of asthmatic women. In particular, sex hormone levels and mediators of bronchial smooth muscle contraction were evaluated.

## Methods

### Human study

#### Study population

To study the relationship between bronchial reactivity and sexual hormones in menstrual cycle, 56 asthmatic pre-menopausal women were recruited from the outpatient clinics of the Respiratory Department of Cava de’ Tirreni Hospital. We recruited women suffering from allergic asthma and documented bronchial hyperreactivity. We excluded women with a history of lung diseases other than asthma, coronary artery disease, congestive heart failure, or cor pulmonale. After accounting for refusals to participate (n = 11), inadequate information on asthma status (n = 3), and non-participation for other reasons (n = 4), 38 women (70.4%) were enrolled in the study. After enrollment, 2 women were excluded because of data missing. A total of 36 females, aged between 23 and 43 years-old (mean: 32.6 ± SD 4.3 yrs-old) completed the study. Among them, 5 were mild smokers with a smoking history of 8.2 ± 2.9 pack/years. No subjects were using oral or intrauterine contraceptives. Obesity, defined as a BMI ≥ 30 kg/m^2^, was present (<35 kg/m^2^) in 5 females. At the time of the study, none of the patients had experienced recent acute exacerbations, and all of them were clinically stable.

Twenty-six (72%) of the subjects had intermittent asthma, due to pollen aeroallergens: six patients (17%) were sensitized to one allergen, twelve patients (33%) were sensitized to two allergens, and eight patients (22%) to three allergens. The remaining ten (28%) women had persistent asthma, mainly due to perennial aeroallergens: five of them (14%) were sensitized to one allergen, and five (14%) were sensitized to two allergens. The mean values of total serum IgE were 449.7 (±336.9) kU/L in the first group (patients with intermittent disease) and 934.2 (±765.8) kU/L in the second group (patients with persistent disease). There was no significant correlation between severity of asthma and RAST classes.

The local Ethics Committee “ASL Salerno” approved the study (n° 357/CdE of September 12, 2012) and all respondents provided informed consent before participation.

At the time of enrollment, women were interviewed and completed a standardized questionnaire that included questions about demographic factors, pregnancy history, health care utilization, smoking history, years of education completed, marital status, asthma history, activity limitations due to asthma, household exposures, asthma-related emergency visits, use of drugs to treat asthma, and other chronic conditions.

#### Measurement of IgE mediated hypersensitivity

The patients underwent intradermal skin testing for IgE-mediated hypersensitivity. Allergy skin-prick tests were carried out using 14 common aeroallergen extracts (Lofarma, Italy): Dermatophagoides (D.) farinae, D. pteronyssinus, cat, dog, ragweed mix, grass mix (Timothy, June, Orchard), ash, beech, birch, hickory, oak, poplar, and the molds Aspergillus and Alternaria tenuis. Histamine (1 mg/mL) and saline (0.9%) solutions were used as positive and negative controls, respectively. Diagnosis of asthma was made on the basis of a history of asthma and the presence of a positive radioallergosorbent test (RAST) for IgE antibodies (RAST-CAP-FEIA, Pharmacia, Uppsala, Sweden). Only women in whom there was an agreement between the results of the skin prick tests and the results of RAST were selected for the study.

#### Pulmonary function test

Patients were asked to refrain from using short-acting ß2-agonists or caffeine-containing beverages for 12 h prior to testing. All enrolled subjects underwent assessment of pulmonary function at rest and measurement of DLCO and airway responsiveness to methacholine as outlined below. These tests were done twice on every subject both during the follicular and luteal phases of the menstrual cycle.

#### Spirometry

Measurements were performed according to American Thoracic Society criteria [12]. Spirometric maneuvers were conducted in triplicate and the highest FEV_1.0_ and FVC values were used in subsequent analyses. Predicted normal values of FEV_1.0_ and FVC were derived from standard equations. SVC was obtained by a slow inspiration from maximal end expiratory lung volume, before flow-volume loop measurement.

#### Lung diffusion

Diffusion capacity for carbon monoxide (DLCO; transfer factor) was obtained by the single-breath method and adjusted for hemoglobin and alveolar volume to yield the diffusion coefficient (KCO). For this purpose, subjects inspired gas atmospheric air mixtures containing 0.3% CH_4_ and 0.3% CO.

#### Cholinergic responsiveness

Cholinergic responsiveness was evaluated by challenging subject with increasing concentrations of inhaled methacoline approved following consensus recommendations. The magnitude of the effect was expressed as percentage of change from the control value (% ΔFEV_1_). Airway reactivity was measured by inhaling increasing concentrations of methacholine from a DeVilbis nebulizer with a breath-synchronized trigger. The initial concentration of 1 mg/ml was progressively doubled until the FEV_1.0_ fell by ≥20% from its original value, and the provocative concentration (PC_20_meth) required to achieve this end point was determined by linear interpolation. The concentrations of methacholine were doubled also above the usual maximum concentration of 25 mg/ml in order to obtain a fall of FEV_1.0_ greater than 20% from the basal value in all subjects. Concentrations of methacholine greater than 25 mg/ml were required in 16 challenges (6 in the follicular phase and 10 in the luteal phase): 2 women needed a concentration greater than the usual maximum only in the follicular phase, 6 women only in the luteal phase, and 4 women in both phases. Bronchodilatation with standard aerosols of albuterol was done in order to reverse cholinergic responsiveness.

#### Hormonal assessments

A venous blood sample was drawn from each participant between 8 AM and 10 AM and serum was obtained by centrifugation. Serum aliquots were then stored at -80°C until analysis.

Prolactin, follicule-stimulating hormone (FSH), luteinizing hormone (LH), testosterone (T), progesterone (PRG) and 17β-estradiol (E2) were measured in all samples using an electrochemiluminescence immunoassay (ElecsysSystem, Roche Diagnostics GmbH, Mannheim, Germany) according to the manufacturer's instructions.

#### Collection and analysis of induced sputum

Induced sputum was collected pre- and post-shift by inhalation of isotonic (0.9%) saline aerosol, in both the follicular and the luteal phases of the menstrual cycle, placed at 4°C and immediately sent to the laboratory. After centrifugation, aliquots of the supernatant fluids were stored at -80°C until further analysis. Cyclic AMP (cAMP) and testosterone levels were measured using Enzyme Immunoassays (Cayman-EIA kit, Michigan, USA). Moreover, PDE activity was evaluated using a PDE assay kit (BPS Bioscience, San Diego, CA). The measurements (PFTs, PC20, and blood collection) were performed on day 1 ± 3 days of menstruation and 14 ± 3 days after the start of menstruation.

### In vitro study

#### PDE activity assay

The ability of testosterone to modulate PDE activity was evaluated by a colorimetric cyclic nucleotide phosphodiesterase assay, according to manufacturer instructions. This assay measures PDE mediated cleavage of cyclic adenosine monophosphate (cAMP) yielding 5’ adenosine monophosphate (5’AMP), which is further cleaved into its nucleosides and phosphate components. The phosphate generated was quantified by a colorimetric reaction with Biomol green. A non-specific PDE inhibitor, 3-isobutyl-1-methylxanthine (IBMX), was included as positive control for inhibitor screening. Physiologically-relevant concentrations of testosterone detected in plasma samples in normal human female subjects (0.3 and 1 nM) were tested and compared to IBMX (40 μM).

### Statistical analysis

#### Statistical analyses for the human study

Group data are expressed as mean (±SD). As our data are generated from small patient populations and these data that are not normally distributed, we used the Mann–Whitney U-test for comparison between non-parametric results. The Spearman's rank correlation coefficient test was used to examine the association between functional data and hormone levels. These analyses were performed using the statistical package SPSS 17.0.

#### Statistical analyses for *in-vitro* evaluations

We performed analyses with two-way ANOVA within groups, followed by the Bonferroni test to correct for multiple comparisons. Data are presented as means ± SEM.

Statistical analyses were performed using GraphPad Prism version 4.7 (GraphPad, San Diego, CA).

Statistical significance was defined as *P <* 0.05.

## Results

### The human study

Among the 36 study subjects studied, 23 had at least one pregnancy with a total of 31 births (with 14 male and 17 female offspring). In 1 out of 14 male births, mothers reported an overall deterioration in symptoms, and in 2 out of 14 male births, mothers reported an improvement in symptoms. However, in 2 out of 17 female births, mothers reported an overall deterioration in symptoms but no mothers of female offspring reported improvement in symptoms. Twelve out of 36 women complained occasionally of perimenstrual asthma, but no patient complained of perimenstrual asthma during the study.

#### PFTs

**Figure 1:**
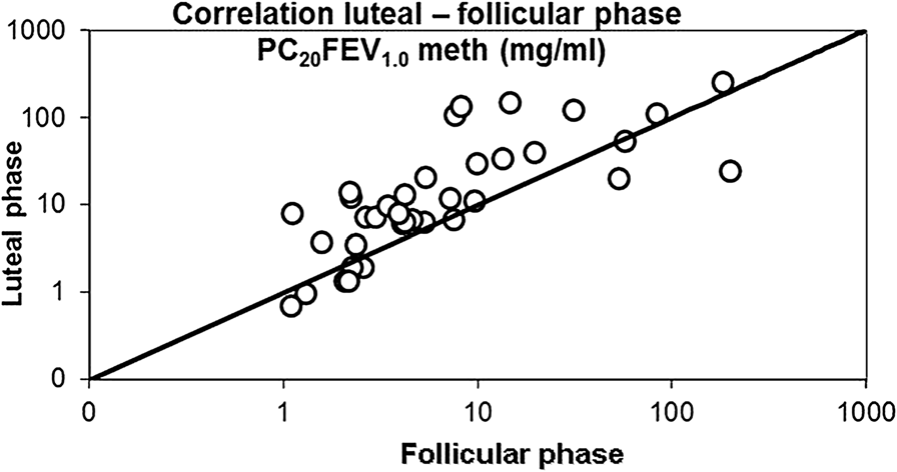
**Correlation between the provocative concentration of methacoline (PC**_**20**_**meth) required to obtain a reduction in FEV**_**1.0**_ **≥ 20% from the baseline value.** On the x-axis: PC_20_meth values measured in the follicular phase; on the y-axis: PC_20_meth values measured in the luteal phase. About 30% of the women had lower PC_20_FEV_1.0_ values during the follicular phase (and vice versa). A logarithmic scale is used on both axes.

Pulmonary function tests showed the absence of restrictive or obstructive defects, as detected by normal total lung capacity with normal FEV_1.0_/FVC ratio and normal DLCO values in both the follicular and the luteal phases of the menstrual cycle in all study subjects. The mean values for PC_20_FEV_1.0_ were not significantly different when measured either in the follicular and the luteal phases (Table 1). However, simple plots of PC_20_FEV_1.0_ values in both follicular and luteinic phases showed that 10 out of 36 (27.8%) of the women had lower PC_20_FEV_1.0_ values during the follicular phase (Figure 1).

**Table 1:** Mean values of pulmonary function tests performed on asthmatic women in the follicular and luteal phases of menstrual cycle

	**FVC**	**FEV**_**1.0**_	**PEF**	**FEF**	**DLCO**	**PC**_**20.0**_
	**% pred.**	**% pred.**	**% pred.**	**% pred.**	**% pred.**	**mg/ml**
**Follicular**	103 ± 13	94 ± 15	78 ± 17	72 ± 33	82 ± 30	21 ± 46
**Luteal**	97 ± 28	91 ± 18	76 ± 21	65 ± 26	81 ± 22	35 ± 56

P > 0,05.

#### Plasma hormones

**Figure 2:**
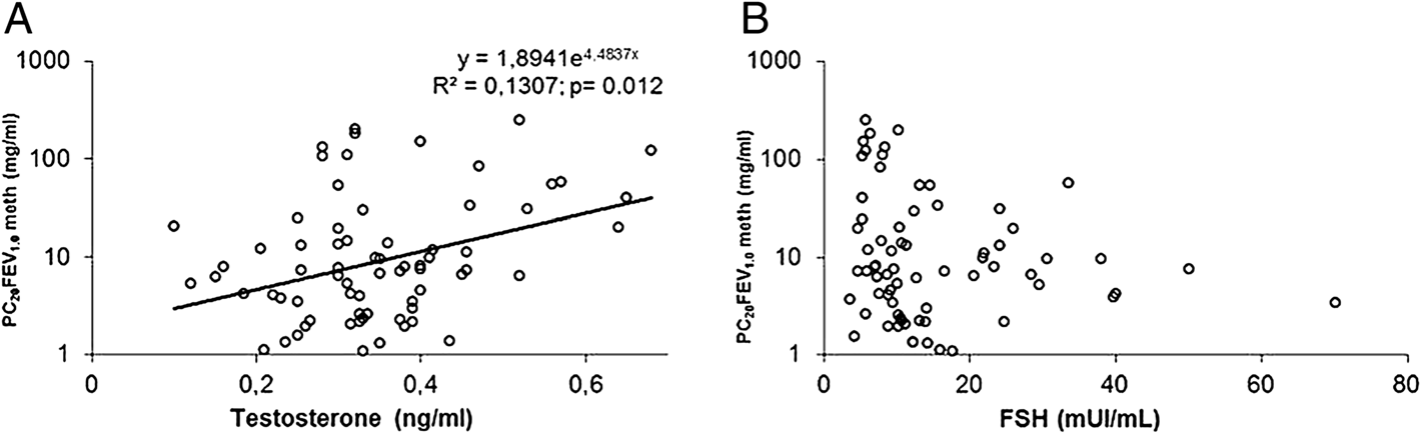
**Relationship in each subject, measured twice (in both the follicular and luteal phases), between serum testosterone (panel A) and FSH (panel B) on the x-axes versus the PC**_**20**_**meth using a logarithmic scale on the y-axes.** Only the relationship between testosterone and PC_20_meth reached statistical significance. P < 0.05.

**Figure 3:**
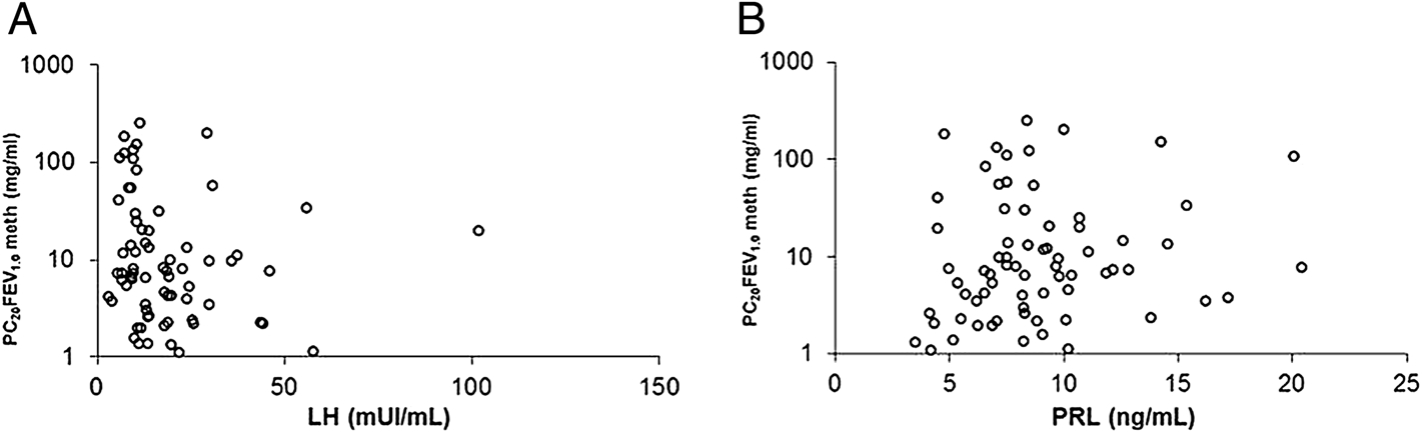
**Relationship in each subject, evaluated twice (in both the follicular and luteal phases), between serum LH (panel A) and prolactin (PRL; panel B) levels on the x-axes versus the PC20meth on the y-axes using a logarithmic scale.** No one of the relationships reached statistical significance. P > 0.05.

**Figure 4:**
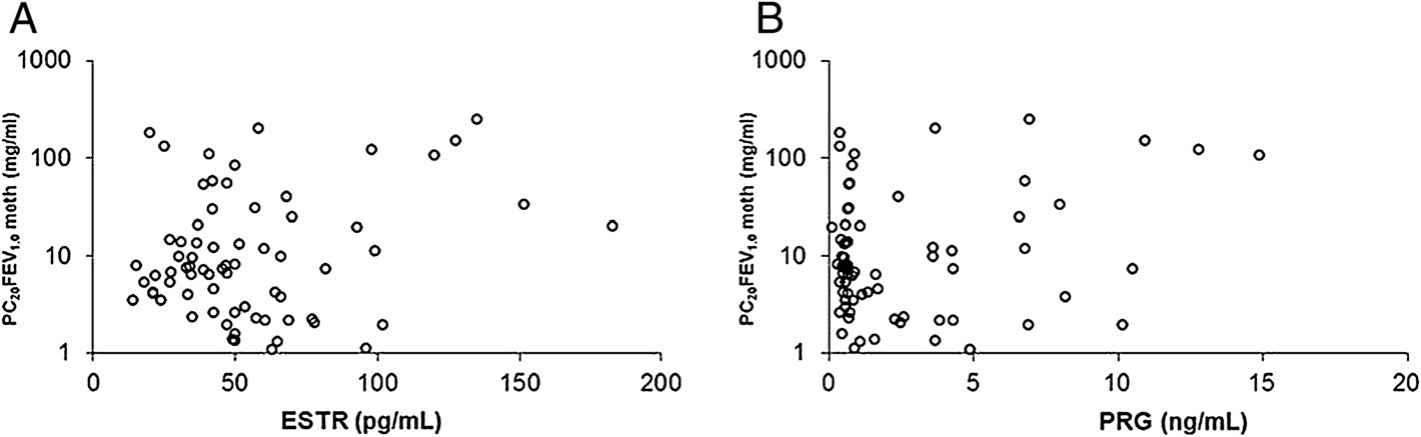
**Relationship in each subject, evaluated twice (in both the follicular and luteal phases), between serum 17β-estradiol (ESTR; panel A) and progesterone (PRG; panel B) levels on the x-axes versus the PC20meth on the y-axes using a in logarithmic scale.** No one of the relationships reached statistical significance. P > 0.05.

Table 2 shows mean values (±1 S.D.) of plasma hormones during the follicular and luteal phases, and Figures 2, 3 and 4 show the relationships between any hormone (independent variable) and PC_20_FEV_1.0_ (dependent variable). Statistically significant differences were obtained only when correlating PC_20_FEV_1.0_ and plasma testosterone levels suggesting that testosterone modulates BR. A stepwise regression analysis, in which all clinical, functional, and hormonal data from both phases of the menstrual cycle were included, identified plasma testosterone levels as the only independent variable (Figure 2A).

**Table 2:** Mean values (±1 S.D) of plasma hormones during the follicular and luteal phases

	**FSH**	**PRL**	**TST**	**LH**	**ESTR**	**PRG**
	**mUI/mL**	**ng/mL**	**ng/ml**	**mUI/mL**	**pg/mL**	**ng/mL**
**Follicular**	18 ± 14	8 ± 3	0,33 ± 0,09	21 ± 12	47 ± 20	1,46 ± 1,57
**Luteal**	12 + 8	10 + 4	0,36 + 0,14	16 + 18	63 + 40	4,15 + 4,40

#### Cyclic AMP (cAMP) levels, testosterone levels and PDE activity in induced sputum

**Figure 5:**
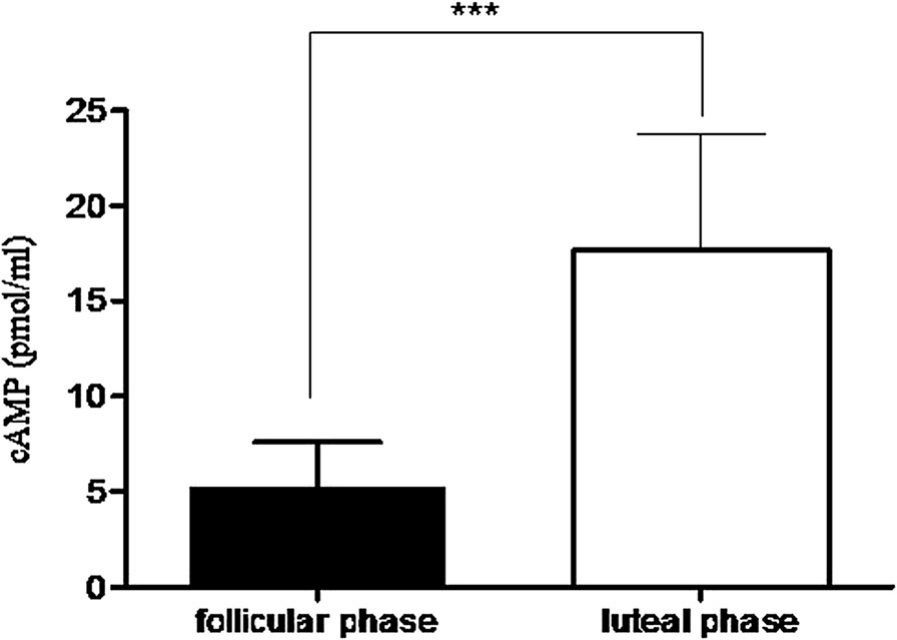
**cAMP levels in induced sputum samples from asthmatic patients.** Sputum cAMP levels were significantly higher in the luteal phase when compared with the follicular phase of the menstrual cycle. Data are mean ± SEM (n = 36). Follicular phase vs luteal phase; ***indicates P < 0.001.

**Figure 6:**
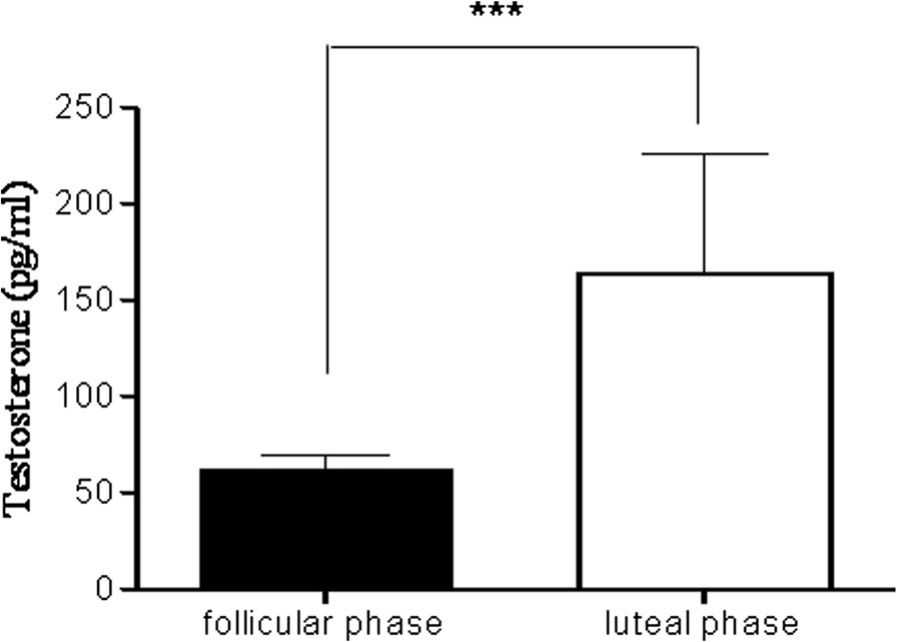
**Testosterone levels in induced sputum samples from asthmatic patients.** Sputum testosterone levels were significantly higher in the luteal phase when compared with levels measured in the follicular phase of the menstrual cycle. Data are mean ± SEM (n = 36). Follicular phase vs luteal phase; ***indicates P < 0.001.

**Figure 7:**
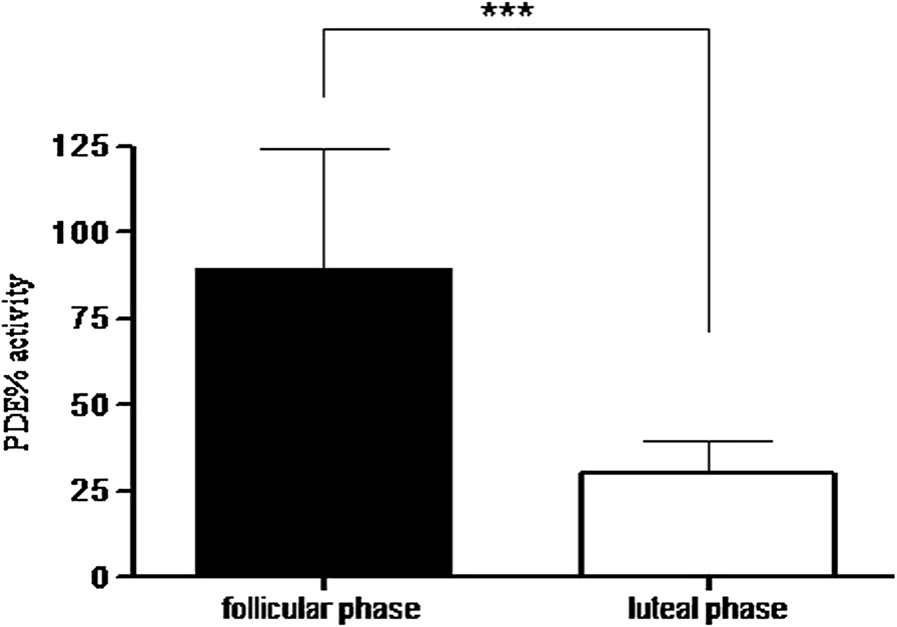
**% PDE activity in induced sputum samples from asthmatic patients.** % PDE activity was significantly higher in the follicular phase when compared with levels measured in the luteal phase of the menstrual cycle. Data are mean ± SEM (n = 36). Follicular phase vs luteal phase; ***indicates P < 0.001.

Cyclic AMP is a key intracellular mediator that contributes to bronchoconstriction in asthmatic airways. Accordingly, we evaluated whether cAMP is present in asthmatic sputum samples and correlates with different phases of the menstrual cycle. As shown in Figure 5, sputum cAMP levels were significantly higher in the luteal phase when compared with the follicular phase of the menstrual cycle. Interestingly, measurement of testosterone levels in the same samples revealed a similar profile, with significantly higher sputum levels of testosterone in the luteal phase compared with the follicular phase of menstrual cycle (Figure 6). Cyclic AMP levels are regulated by phosphodiesterases, which hydrolyze and thereby, reduce cAMP levels in cells. Thus, we measured PDE activity in sputum samples from the our asthmatic patients. Sputum PDE activity showed a different trend when compared with to those of cAMP and testosterone, as they were lower in the luteal phase when compared with sputum PDE activity levels measured in the follicular phase of menstrual cycle (Figure 7).

### In vitro study

#### Testosterone does not reduce PDE activity in vitro

**Figure 8:**
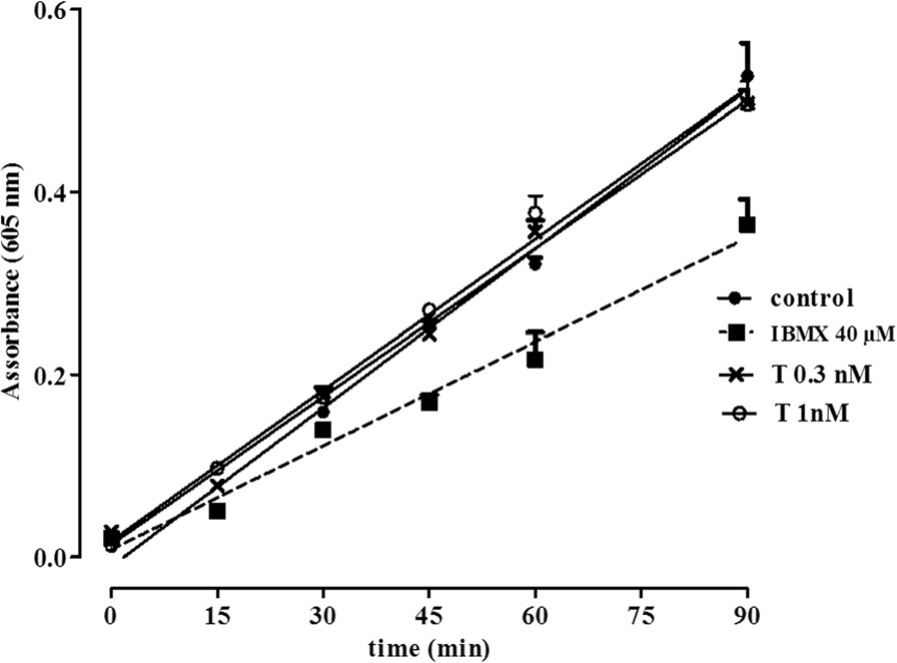
**Testosterone does not affect PDE activity.** PDE activity (cyclic nucleotide-degrading PDE activity) was evaluated in the presence of the indicated concentration of testosterone or IBMX, using a commercially available cyclic nucleotide phosphodiesterase kit. IBMX is a non selective PDEs inhibitor, used as positive control. P > 0.05.

Next, we assessed whether testosterone directly reduces PDE activity to thereby increase cAMP levels in airway samples from patients with asthma. To assess this possibility, we incubated cell-free, purified PDE with or without two different concentrations of testosterone in vitro, and measured residual PDE activity. As shown in Figure 8, testosterone had no direct effect on PDE activity at either concentration tested. Thus, it is unlikely that testosterone modulates cAMP levels in asthmatic airways by directly interfering with PDE activity.

## Discussion

Perimenstrual worsening of asthma with an increase in symptoms and significant decline in peak flow (20%) has been reported in 30-40% of women [13]. Our study, carried out on 36 asthmatic women, has documented that 10 out of 36 (27.8%) of them showed a trend towards a lower PC_20_FEV_1.0_ in the follicular phase when compared with their response in the luteal phase (or vice versa). However, this result was not statistically significant due to the high degree of variability between subjects, and was not clinically evident in terms of asthma-related symptoms. In this study, we have also demonstrated that in the luteal phase of menstrual cycle the mean level of testosterone in both sera and induced sputum samples is increased when compared with mean levels measured in the follicular phase. Higher testosterone levels in sputum samples were, in turn, associated with higher cAMP levels and lower PDE activity in sputum samples during the luteal phase suggesting that testosterone regulates the generation of cAMP by reducing PDE activity.

A few studies have focused on bronchial hyperresponsiveness to various constrictor agents during the menstrual cycle in asthmatic women [8,9,13-18], but no cyclical variations were found in the subjects’ responses to methacholine or histamine challenges [8,9,13,14]. However, two studies by Tan et al. [15,16] showed an increase in PC_20_ in response to adenosine monophosphate challenges in the luteal phase when compared with responses in the follicular phase. This could indicate an effect of female sex hormones on airway inflammation during the menstrual cycle, as changes in AMP closely reflect changes in eosinophilic airway inflammation [17-19].

Women of reproductive age experience cyclic variations in serum concentrations of sex hormones [20]. During the 4 days after menstruation, FSH, LH, 17-β-estradiol, progesterone, and testosterone levels are low. During the follicular phase of the menstrual cycle, progesterone and testosterone serum levels remain low, while levels of FSH and LH peak. Finally, during the luteal phase, FSH and LH levels are low, whereas 17-β-estradiol, progesterone, and testosterone are moderately high [20].

Clinical observations indicate that the hormonal fluctuations described above might be responsible for the cyclic changes in asthmatic symptoms reported by patients [5,7,21,22]. Our study adds changes in testosterone levels to the known hormonal fluctuations that have been demonstrated during the menstrual cycle. Our study documented a significant positive correlation between PC_20_FEV_1.0_ and serum testosterone levels during the follicular phase. In contrast, plasma levels of other sex hormones that we measured did not correlate with PC_20_FEV_1.0_. Moreover, we found significantly higher testosterone levels in induced sputum of our patients during their luteal phases when compared with testosterone levels measured in the follicular phases of their menstrual cycles.

Patients presenting premenstrual asthma worsening are frequently affected by alterations of the cyclic changes in serum levels of progesterone [20,23]. Progesterone, like all other steroid hormones, is synthesized from pregnenolone. Progesterone is the precursor of aldosterone, which in turn can be converted to testosterone [24]. It has been suggested that low testosterone levels may significantly alter immune responses and airway smooth muscle reactivity [25], through genomic or non-genomic mechanisms. Wulfsohn et al., treated female asthmatic patients with different hormones including testosterone and demonstrated an improvement in symptoms in ~90% of them showing an improvement in about 90% of them [26]. Furthermore, Mileva et al., found low levels of testosterone in blood samples from patients with severe and moderately severe asthma when compared with patients with mild asthma [27]. In our study, in addition to higher testosterone levels, we also documented significantly higher cAMP concentrations and lower PDE activity in induced sputum samples from patients in the luteal phase when compared with PDE activity measured during the follicular phase. PDEs are a diverse family of enzymes that play a key role in reducing levels of the second messenger, cAMP, and hence bronchial smooth muscle tone. Thus, the relatively low PDE activity in sputum samples from patients during the luteal phase of the menstrual cycle may contribute to the increased airway levels of cAMP in these subjects in their luteal phase and vice versa for the follicular phase. Although sputum testosterone levels correlated indirectly with sputum PDE activity during the various phases of the menstrual cycle, our in vitro results exclude the possibility that testosterone directly inhibits the catalytic activity of PDE. It is possible that an as-yet-unidentified testosterone-induced factor inhibits PDE activity in the airways of female asthmatics during the luteal phases of their menstrual cycles. In previous studies, we demonstrated that vasodilator effect of testosterone involves H2S, a novel gaseous mediator [28], which, in turn, may act as an endogenous inhibitor of PDE activity [29]. Therefore, we speculate that H2S mediates the inhibitory effect of testosterone on PDE activity in the airways of PMA subjects.

In rabbits, testosterone deficiency induced by castration reduced trabecular smooth muscle content, and this reduction was restored by testosterone treatment [30]. Moreover, the ability of testosterone to regulate PDE expression in rat corpus cavernosum has also been demonstrated [31]. In a human study, Aversa et al. investigated the role of androgens in regulating trabecular smooth muscle relaxation in the corpus cavernosum in men with erectile dysfunction in response to vasoactive challenge [32]. The findings indicated that, in men with erectile dysfunction, low testosterone levels correlate with impaired relaxation of cavernous endothelial and corporeal smooth muscle cells independently of age. These findings provide some clinical evidence that androgens regulate smooth muscle function in vivo. Finally, reduced responsiveness to β2-agonists has been observed in surgically removed bronchial tissue from asthmatic patients [33]. In addition, testosterone promotes smooth muscle relaxation in preparations of bronchial tissue isolated from asthmatic animals with desensitized beta-adrenoceptors [34] and potentiates isoprenaline-mediated relaxation of bronchial smooth muscle [35]. Taken together, these studies suggest that smooth muscle tone in different tissues (trachea, bronchi, penis and the vasculature) is at least partially androgen-dependent.

The interpretation of our results should take into account the existing experimental and clinical data showing that female sex hormones influence lung function, airway responsiveness, and inflammation, but their effects vary depending on the experimental models and the end points analyzed [36].

## Conclusions

In conclusion, our data provide evidence that variations in testosterone levels during the menstrual cycle may contribute to fluctuations of PC_20_FEV_1.0_ during the menstrual cycle of premenopausal women. Obviously, this is a biologic phenomenon that might not even have automatically clinical implication, as demonstrated by absence of perimenstrual asthma in our population during the study. Our results suggest that additional studies are needed to assess whether targeting testosterone and/or cAMP in asthmatic patients have potential to improve the management of the worsening of asthma during the menstrual cycle.

## Abbreviations

PMA: Perimenstrual asthma; BR: Bronchial reactivity; DLCO: Diffusing capacity of the lung for carbon monoxide; cAMP: Cyclic adenosine monophosphate; PDE: Phosphodiesterase; FEV_1.0_: Forced expiratory volume in one second; FVC: Forced vital capacity.

## Competing interests

The authors declare that they have no competing interests.

## Authors’ contributions

CS and MP performed the recruitment of patients and functional clinical study. GS, NS and MM performed assays of hormone levels in induced sputum and plasma. F Roviezzo and MRB performed the in vitro study of PDE activity. F Rossi and CAO made substantial contributions to the final version to be published. MM, FP, MP and BD made substantial contributions to conception, design and drafting the article. All authors read and approved the final manuscript.

## Pre-publication history

The pre-publication history for this paper can be accessed here:

http://www.biomedcentral.com/1471-2466/14/108/prepub
